# Muscle glycogen metabolism changes in rats fed early postnatal a fructose-rich diet after maternal protein malnutrition: effects of acute physical exercise at the maximal lactate steady-state intensity

**DOI:** 10.1186/1758-5996-6-118

**Published:** 2014-11-06

**Authors:** Lucieli T Cambri, Carla Ribeiro, José D Botezelli, Ana C Ghezzi, Maria AR Mello

**Affiliations:** São Paulo State University (UNESP), Rio Claro, SP Brazil; Federal University of Mato Grosso (UFMT), Cuiabá, MT, Brazil. Physical Education Department, UFMT, Av. Fernando Corrêa da Costa, 2367 - Boa Esperança, Cuiabá, Zip-Code:- 78060-900 MT Brazil

## Abstract

**Background:**

The objective was to evaluate the muscle glucose metabolism in rats fed a fructose-rich diet after fetal protein malnutrition, at rest and after acute physical exercise at maximal lactate steady-state intensity.

**Methods:**

The male offspring born of mothers fed on a balanced or low-protein diet were split in four groups until 60 days: Balanced (B): balanced diet during the whole period; Balanced/Fructose (BF): balanced diet in utero and fructose-rich diet after birth; Low protein/Balanced (LB): low-protein diet in utero and balanced diet after birth; Low protein/Fructose (LF): low protein diet in utero and fructose-rich diet after birth.

**Results:**

Acute physical exercise reduced the muscle glycogen concentrations in all groups, although the LF group showed higher concentrations at rest. There was no difference among the groups in the glucose uptake and oxidation rates in the isolated *soleus* muscle neither at rest nor after acute exercise. However, glycogen synthesis was higher in the LF muscle than in the others at rest. Acute physical exercise increased glycogen synthesis in all groups, and the LF group showed the highest values.

**Conclusion:**

The fructose-rich diet administered in rats after fetal protein malnutrition alters muscle glycogen concentrations and glycogen synthesis in the rest and after acute exercise at maximal lactate steady-state intensity.

## Background

According to epidemiological data, metabolic syndrome has been associated to the increasing in the mortality caused by cardiovascular problems in 30 to 400%, depending on the studied population, the adopted definition of syndrome and the type of study [1]. It has estimated that the prevalence of metabolic syndrome, in the United States, is around 25 to 29% of the adult population and 50 to 60% of the population over 60 years old. In 2000, nearly 47 million residents of that country presented metabolic syndrome [2]. In developing countries, the prevalence of metabolic syndrome in adolescents has increased substantially, especially when it analyzes overweight and obese adolescents [3].

There are controversies in the identification of metabolic syndrome in humans, since there are many international standards, which mainly differ the cut points for abnormality in each parameter used, which consequently originates contradictions in diagnostic rate in the studies about the theme. There are clinical and epidemiological evidences displaying the association between the ingestion of fructose, vastly used as sweetener in soft drinks and food, and the appearance and development of metabolic syndrome markers [4]. Experimentally, signs of metabolic syndrome, such as hypertension, hyperinsulinemia, hypertriglyceridemia and insulin resistance can be triggered in adult rodents by a fructose-rich diets [5, 6]. On the other hand, there are some evidences that intrauterine malnutrition might “program” the fetal tissues making them more vulnerable to nutrition-related disorders, such as type 2 diabetes, metabolic syndrome and other chronic diseases in adulthood [7]. Therefore, it is important to evaluate if organisms subjected to early malnutrition are more susceptible to the negative metabolic effects of the excess of fructose in the diet.

In this sense, animal models, using controlled conditions, can contribute to investigate this issue. To develop experimental protein deficiency, isocaloric diets of low protein content are often used [8, 9].

Most studies in animal models analyze the influence of early protein malnutrition in the absence of physical activity or exercise. Likewise, nutritional recovery is usually obtained with balanced diets and the absence of physical exercise [8]. Some studies have shown favorable effects of chronic physical exercise in the nutritional recovery of malnourished children [10] and rats [9]. Similarly, although no consensus about the chronic effects of physical exercise on the metabolic syndrome, its benefits are clear on the isolated pathologies, such as obesity [11], diabetes mellitus [12], dyslipidemia [13] and hypertension [14]. However, oftentimes the studies about metabolic parameters and physical exercise in animal models are criticized due to the lack of information about the intensity of effort performed during the physical exercise. Therefore, it is very interesting to analyze the glucose metabolism after acute physical exercise performed at the maximal lactate steady-state, because this intensity can be used as a standard to exercises prescription and to improve the disturbances related the metabolic syndrome, and also to verify the effects of physical training in the nutritional rehabilitation. In this exercise intensity, both glycolytic and oxidative pathways contribute to the energy production.

Still, data about the acute effects of physical exercise in organisms recovering from protein malnutrition and/or fed on a fructose-rich diet are limited. In a recent study from our laboratory [5], it was analyzed blood variables and it was observed that, most responses to acute physical exercise were not influenced by early malnutrition and/or by the fructose overload.

Therefore, the objective of this work is to evaluate the muscle glucose metabolism in young rats fed a fructose-rich diet after fetal protein malnutrition, at rest and after an acute bout of swimming exercise at the maximal lactate steady-state intensity.

## Method and materials

### Animals and diets

Twenty pregnant adult (90-day-old) rats norvegicus albinus (Wistar), were kept in individual cages, in a room with a controlled temperature (25°C), with a photoperiod of 12 hours light and 12 hours dark, with lights on from 06:00 to 18:00 hours and given them free access to water and food during the whole experiment. The cages were changed 4 days/week during the experimental period. All procedures adopted with the animals were approved by the Committee of Ethics in Animal Research, State University of Campinas (UNICAMP; Campinas, São Paulo, Brazil), under protocol n° 1487–1.

The rats were fed balanced (17% protein), low-protein (6% protein), and fructose-rich (60% fructose) diets, according to the composition described on Table 1.

**Table 1:** **Composition of the diets**

Components (g/kg diet)	Balanced ^1^	Low-protein ^2^	Fructose ^3^
**Casein (84% protein)** ^**4**^	202	71.5	202
**L-cystine**	3	1	3
**Starch**	397	480	-
**Dextrin**	130.5	159	-
**Sucrose**	100	121	27.6
**Fructose**	-	-	600
**Mineral mix (AIN-93GMX)** ^**1**^	35	35	35
**Vitamin mix (AIN-93GVX)** ^**1**^	10	10	10
**Soybean oil**	70	70	70
**Fiber**	50	50	50
**Choline hydrochloride**	2.5	2.5	2.5

^1^According to the American Institute of Nutrition (AIN-93G) [15]. The Mineral mix and Vitamin mix contained 22.10 and 97.46% of sucrose, respectively. For detailed composition, see Reeves et al. [15].

^2^According to Latorraca et al. [7].

^3^According to Cambri et al. [6].

^4^Values corrected according to the amount of protein in the casein.

To calculate caloric content in these three diets was considered as carbohydrate: starch, dextrin, sucrose and sucrose contained in mineral mix and vitamin mix; as protein: casein, with 84% of protein and L-cystine; and as fat: soybean oil. The low-protein diet differed from the balanced diet in the contents of protein and carbohydrate. The fructose-rich diet had the same macronutrient content as the balanced diet except in the carbohydrate composition, the starch and dextrin were changed by fructose. The estimate of caloric content was based on the standard physiological fuel mean values for carbohydrate, protein and fat of 4, 4 and 9 kcal, respectively. So, all diets had approximately 3,9 kcal/g, with the balanced diet and fructose-rich diet containing 66.1% carbohydrate; 17.7% protein and 16.2% lipids, and the low protein diet containing 78.1% carbohydrate; 6.1% protein and 15.8% lipids in relation to total caloric content.

### Experimental groups

**Figure 1:**
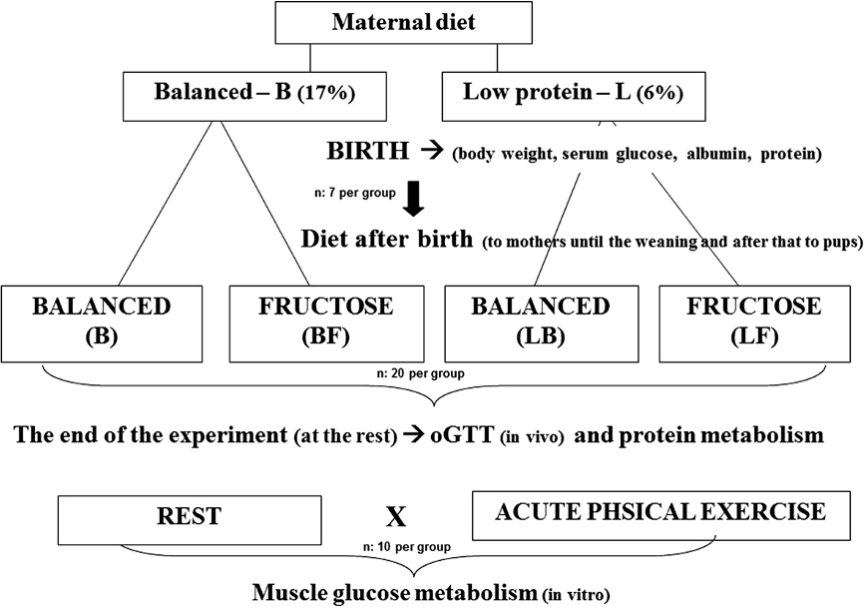
**Experimental design.**

The animals were split into two groups, according to the diet received during pregnancy: balanced (B) - diet containing 17% protein and low-protein (L) - diet containing 6% protein. The experimental design is showed in Figure 1.

On day of birth, seven male B neonates and seven male L neonates were weighed and killed to analyze the serum glucose, total protein, and albumin concentrations [16], to confirm the installation of protein malnutrition in the L group. The remaining offspring were distributed into four groups (n =20 per group) according to the diet they received until 60 days of age. The litters were adjusted until each female fed only 8 offspring. In some cases the female neonates were excluded to facilitate this adjustment. So, the diet after birth was given to mothers until the weaning and after that to pups. After birth of the puppies, all diets contained 17% protein. After weaning, only the male rats were maintained in the study. Rats were maintained in collective plastic cages (5 rats/cage). Until weaning (from birth to 21 days), the mothers received corresponding diets:

- ● Balanced (B): a diet containing 17% protein during the whole experiment.
- ● Balanced/fructose (BF): a diet containing 17% protein in utero and a fructose-rich diet (60% fructose) from birth until 60 days of age.
- ● Low protein/balanced (LB): a diet containing 6% protein in utero and a diet with 17% protein from birth until 60 days of age.
- ● Low protein/fructose (LF): a diet containing 6% protein in utero and a fructose-rich diet (60% fructose) from birth until 60 days of age.

All rats had their body weight registered once a week, from weaning (21 days) until 60 days of age.

### In vivo analysis

#### Oral glucose tolerance test – oGTT

The oGTT was performed with the rats at 60 days of age, after a 12 hours fasting. A glucose solution at 20% (2 g/kg of body weight) was administered to the rats through a gastric polyethylene probe. Blood samples were collected 0, 30, 60 and 120 minutes after the glucose administration, through a small cut on the extremity of the rat’s tail, to determine the glucose concentrations. The blood glucose concentrations were determined by the glucose oxidase-peroxidase enzymatic colorimetric method, using commercial kits (LABORLAB®, Guarulhos, São Paulo/Brazil) and area under a curve of glucose (AUC_glucose_) were calculated by the trapezoidal method, using the Microsoft Excel 2007® software.

### Evaluation of the maximal lactate steady-state test

The evaluation of the maximal lactate steady-state test was realized to determine the overload to a single session of swimming. The rats were adapted to the water for 10 days, with the objective of reducing the stress caused by the physical exercise performed in this environment. The water temperature was always kept at 31 ± 1°C. The adaptation began with five, ten and fifteen minutes in shallow water; moving to five, ten and fifteen minutes in deep water; and subsequently leading to five minutes with a bag tied to the thorax and five, ten and fifteen minutes with a bag tied to the chest containing an overload of 3% body weight.

The maximal lactate steady-state during swimming was determined, through the application of overloads, which varied from 6.5 to 8.5% of body weight for 25 minutes, on alternate days, with sampling of blood from the tail extremity every 5 minutes for lactate [17] dosage by the enzymatic method. The maximal lactate steady-state was considered the highest working load in which the difference in blood lactate concentrations, between the 25th and 10th minutes of exercise, was equivalent to, or lower than, 1 mmol.L^−1^ [18].

The maximal lactate steady-state is defined as the highest lactate concentration and work load that can be sustained, during exercise sessions with fixed loads, without the continuous accumulation of blood lactate [18]. The load associated with the maximal lactate steady-state is frequently used for training prescription and evaluation of aerobic conditioning [19]. So, the maximal lactate steady-state protocol was used to identify the threshold of transition between aerobic and anaerobic metabolism.

### Sample collection

Forty eight hours after the last evaluation “in vivo”, half of the each group (n: 10 rats), were anesthetized in a carbon dioxide chamber and then euthanized at rest by decapitation, 48 hours after the last evaluation “in vivo”. The remaining was, immediately before euthanasia, submitted to a single session of swimming exercise for 20 minutes, with an overload equivalent to the maximal lactate steady-state.

### Muscle protein synthesis

Longitudinal strips from the *soleus* muscle (70 mg) were preincubated for 30 minutes in a RPMI 1640 medium (with glutamine and without red phenol and sodium bicarbonate) that was supplemented with fat-free 0.1% bovine serum albumin (BSA) and 100 μU ml^−1^ insulin and saturated with a 95% O2/5% CO_2_ gas mixture. The strips were then, transferred to fresh RPMI medium, which contained the same supplements in addition to 0.05 ACi mL^−1^ [14C] phenylalanine, and incubated for two more hours. At the end of this incubation, the muscle strips were homogenized in 5% trichloroacetic acid (TCA) and centrifuged at 2000 rpm for 15 minutes at 4°C. TCA-insoluble material was washed three times with 5% TCA. The resulting precipitate was dissolved for 30 minutes in 10% sodium dodecyl sulfate at room temperature to determine both protein content and the radioactivity incorporated into the muscle protein. Muscle protein content was determined by the folin-phenol method, and the radioactivity incorporated into the muscle proteins was determined with a liquid scintillation counter. Protein synthesis was calculated by dividing the incorporated radioactivity by the specific phenylalanine radioactivity in the incubation medium [20]. This parameter was analyzed only in animals at rest.

### Protein degradation

Tyrosine liberation by isolated muscles in the presence of cyclohexamide was employed as the protein degradation index [20]. This method is based on the fact that the amino acid tyrosine is neither synthesized nor degraded by skeletal muscle. Longitudinal strips from the *soleus* muscle (70 mg) were preincubated in Krebs–Ringer buffer (NaCl, 1.2 mmol/L; KCl, 4.8 mmol/L; NaHCO_3_, 25 mmol/L; CaCl_2_, 2.5 mmol/L; KH_2_PO_4_, 1.2 mmol/L; MgSO_4_, 1.2 mmol/L; pH 7.4) that was supplemented with 5.5 mmol/L glucose, 1.34% BSA, 5 AUmL^−1^ insulin, and 5.0 mmol/L cyclohexamide and saturated with a 95% O_2_/5% CO_2_ gas mixture. Subsequently, the muscle strips were transferred to fresh medium with the same [20].

### Serum analyses

Blood samples were collected immediately after euthanize for total protein and albumin determinations by colorimetric methods (LABORLAB® kits, Guarulhos, São Paulo/Brazil).

### Muscle glucose metabolism

The *soleus* muscle of the left paw was excised with minimum lesion for the evaluation of the metabolism of glucose. Longitudinal slices of the muscle (25-35 mg) were placed in scintillation flasks with a 20 mL capacity siliconyzed, containing 1.5 mL of Krebs-Ringer bicarbonate buffer (The Krebs-Ringer buffer, base in the pre-incubation and incubation mediums, was composed of: NaCl 0.6%, NaHCO_3_ 0.19%, HEPES 6.64 mM, KCl 0.032%, CaCl_2_ 1.14nM, KH_2_PO4 0.015%, MgSO_4_ 0.03%. This solution was gassed for 20 to 30 minutes in O_2_/CO_2_ (95%/5%) and the pH was adjusted to 7.4. To this solution, 20 volumes of bovine fat-free serum albumin were added. Sodium pyruvate was added to the incubation medium for a concentration of 5 mM. Glucose (5.5 mM) was added to the incubation medium containing [U-^14^C] glucose (0.25 μCi/mL), [^3^H] 2-deoxyglucose (2DG = 0.5 μCi/mL) and insulin (100 μUl/mL). With the additions, the pH was adjusted to 7.4 and the mediums transferred to the flasks that were sealed and balanced in bath at 37°C under gassing in O_2_/CO_2_ (95%/5%) for at least 15 minutes). The flasks were closed with rubber stoppers, sealed with a plastic ring and subjected to 30 minutes of pre-incubation with agitation in Dubnoff bath at 60 rpm and continuous gassing with O_2_/CO_2_ (95%/5%). After this period, the muscle slices were transferred to new scintillation flasks (external flask) within which small shell-shaped tubes (internal flask) were installed with a straight rod, approximately 3 cm long, inserted in the rubber stoppers of the external flasks.

Each external flask contained 1.5 mL of Krebs-ringer buffer and each internal flask, 700 μl of hyamine 10×. After 60 minutes of incubation in this system, with gassing during the first 15 minutes, 100 μl of trichloroacetic acid (TCA) 25% were added to the external flask, for the liberation of CO_2_. The preparation was maintained for over 3 hours in the system, but with the muscle slice separated from the TCA solution. Afterwards, 200 μl of the liquid contained in the internal flask were removed to determine the CO_2_ produced. The muscle slice was immediately digested in 0.5 mL of KOH for the extraction [21] and dosage of muscle glycogen [22]. The pre-incubation and incubation temperatures were at 37°C. Non incubated slices of the same muscle were also, used with similar weight of those incubated, to determine the glycogen concentration [22].

Glucose uptake was evaluated using a 2 DG as marker, and incorporation of the ^14^C to glycogen (synthesis), measuring the ^3^H radioactivity of the 2 DG and the ^14^C radioactivity of the glucose, respectively, with the beta particle counter. To estimate the oxidized glucose (CO_2_ production, the ^14^C radioactivity was determined in the liquid (hyamine) collected from the internal flask of the incubation system, with aid of a beta particle counter.

### Statistical analysis

Student’s *t* test was used for comparisons between the newborn groups. At the end of the experiment, two-way ANOVA was used for comparisons among the groups (inter-group), both at rest and after acute physical exercise, followed by Newman-Keuls’ post hoc test when necessary. Student’s *t* test was used for intra-group comparisons as to the effect of acute physical exercise. A p value of <0.05 was considered to be statistically significant.

## Results

At birth, the low-protein diet in fetal period caused a statistically significant (p < 0.05) decrease in body weight (B: 6.49 ± 0.83; L: 5.31 ± 0.79 g), serum glucose (B: 81 ± 11; L: 65 ± 16 mg/dL), albumin (B: 3.36 ± 0.19; L: 1.29 ± 0.19 mg/dL) and protein (B: 7.45 ± 1.82; L: 3.94 ± 1.81 mg/dL).

**Figure 2:**
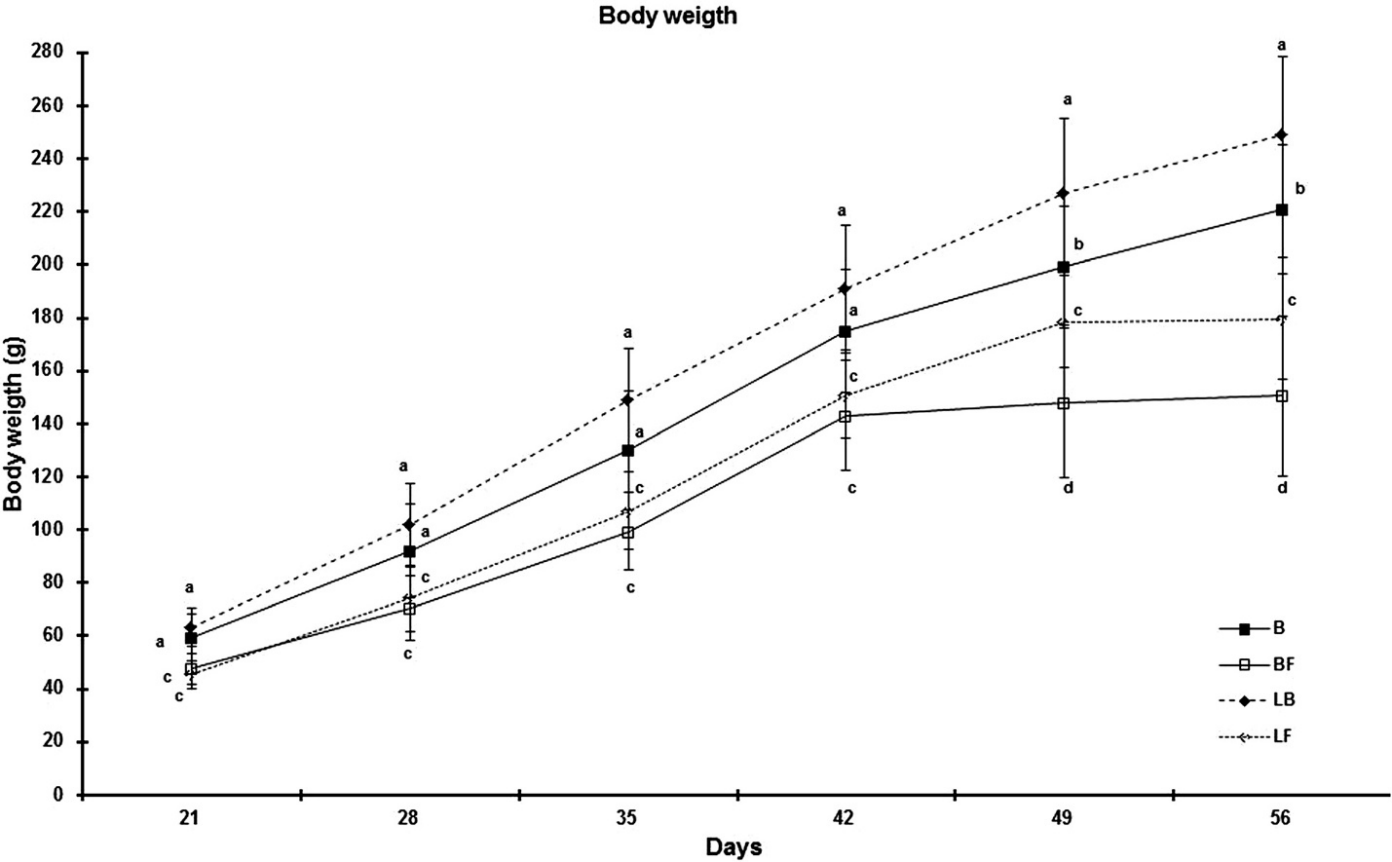
**Body weight of the rats from weaning (21 days) until the end of the experiment (60 days).** Results are expressed as means ± standard deviation of 10 animals per group. B, balanced; BF, balanced–fructose; LB, low protein–balanced; LF: low protein–fructose. Different letters indicate a statistically significant difference (a#b#c#d) among groups (p <0.05).

The diet adopted after birth (fructose-rich or balanced), but not in the fetal period (low protein or balanced) seems to have acute negative influence in the weight gain. Body weight in groups fed on fructose-rich diet (BF and LF) was smaller than groups fed on balanced diet (B and LB). Indeed, the body weight in those groups fed balanced diet during the nutritional recovery period (B and LB) was similar until 42 days, as the groups fed fructose-rich diet after birth (BF and LF) was similar until the same period. However, in long time (after 42 days), the interaction between diets in the fetal period and after birth exerted influence among all groups. After this point, BF group showed the lowest body weight when compared to other groups (Figure 2).

**Figure 3:**
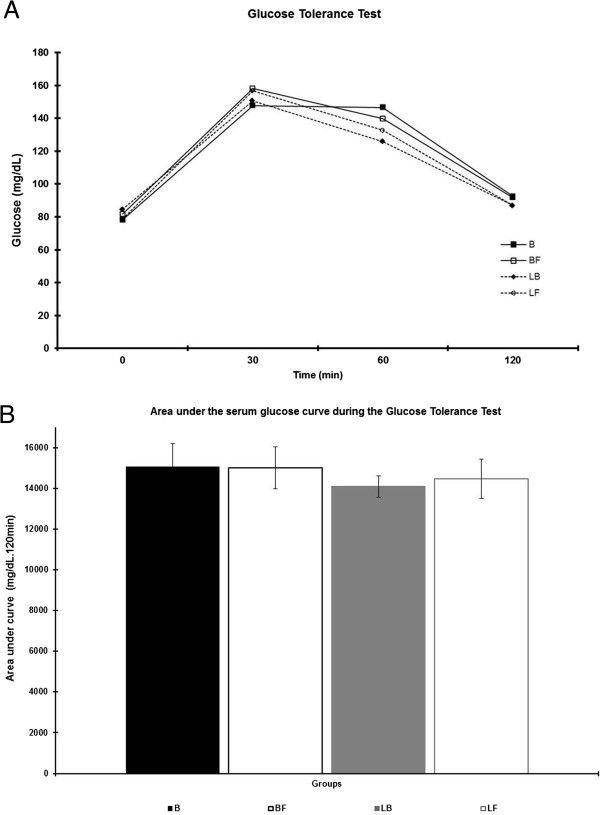
**Blood glucose during the glucose tolerance test at the 60 days. (A)** Serum glucose (mg/dL). **(B)** Area under the serum glucose curve (mg/dL.120 min). Results expressed as means ± standard deviation of 10 animals per group.

Serum glucose levels (Figure 3A) and the AUC_glucose_, determined by the glucose values (Figure 3B) during the glucose tolerance oral test, were similar in all groups.

At the end of the experiment, the serum albumin (B: 3.86 ± 0.25; BF: 3.84 ± 0.23; LB: 4.19 ± 0.27; LF: 4.01 ± 0.58 mg/dL) and total protein (B: 5.51 ± 0.12; BF: 5.59 ± 0.08; LB: 5.51 ± 0.28; LF: 5.78 ± 0.16 mg/dL) parameters, altered at birth by the fetal protein restriction, were reestablished, independently of the diet adopted during nutritional recovery. Therefore, there was no difference among the groups for these parameters. Additionally, there is not significantly difference among the groups in the protein synthesis and nor in the degradation in the *soleus* muscle (Table 2).

**Table 2:** **Protein synthesis (pmol/mg.h) and degradation (pmol/mg.h) in the** ***soleus*** **muscle at the 60 days**

	B	BF	LB	LF
**Protein synthesis**	12.5 ± 4.9	19.9 ± 6.9	13.0 ± 4.4	13.9 ± 4.4
**Protein degradation**	178.2 ± 48.1	205.6 ± 53.9	163.5 ± 38.8	141.1 ± 30.4

Results expressed as the mean ± standard deviation of 10 rats per group.

B: balanced; BF: balanced/fructose; LB: low protein/balanced; LF: low protein/fructose.

There was no difference (P < .05) among groups by Two-Way ANOVA.

The fructose overload after fetal protein malnutrition (LF) increased (p < 0.05) the muscle glycogen content. However, the session of acute physical exercise decreased (p < 0.05), in all groups, the muscle glycogen concentrations, which did not differ among themselves after the session of physical exercise (Table 3).

**Table 3:** **Muscle glycogen and glucose uptake, glucose oxidation and glycogen synthesis in the isolated** ***soleus*** **muscle in the rest (R) and after acute physical exercise (E) at the 60 days**

		B	BF	LB	LF
**Concentration of glycogen (mg/100 mg)**	**R**	0.44 ± 0.06^a^	0.53 ± 0.11^a^	0.51 ± 0.09^a^	0.65 ± 0.17^b^
	**E**	0.28 ± 0.09*	0.27 ± 0.09*	0.26 ± 0.09*	0.29 ± 0.15*
**Glucose uptake (μmol/g.h)**	**R**	2.60 ± 0.17	2.80 ± 0.42	2.38 ± 0.47	2.22 ± 0.65
	**E**	2.26 ± 0.41	2.50 ± 0.38	2.19 ± 0.32	2.27 ± 0.47
**Glucose oxidation (μmol/g.h)**	**R**	4.09 ± 0.72	3.58 ± 1.64	3.40 ± 1.12	3.41 ± 1.95
	**E**	3.91 ± 1.20	4.08 ± 1.60	3.18 ± 0.47	3.33 ± 1.70
**Glycogen synthesis (μmol/g.h)**	**R**	0.03 ± 0.01^a^	0.04 ± 0.02^a^	0.04 ± 0.03^a^	0.07 ± 0.01^b^
	**E**	0.08 ± 0.02^a^*	0.06 ± 0.04^a^	0.08 ± 0.04^a^*	0.18 ± 0.13^b^*

Results expressed as the mean ± standard deviation of 10 rats per group.

B: balanced; BF: balanced/fructose; LB: low protein/balanced; LF: low protein/fructose.

R: at rest; E: after acute physical exercise.

Different letters indicate significant difference among groups (a#b). Two-Way ANOVA and Newman-Keuls’ Post-Hoc (p < 0.05).

*intra-group difference by Student’s t-test (at rest *vs.* acute physical exercise).

The data on glucose metabolism in the *soleus* muscle are presented on Table 3. Glucose uptake and oxidation were analyzed, as well as glycogen synthesis. The fructose-rich diet after fetal protein malnutrition (LF) caused an increase in the glycogen synthesis by the *soleus* muscle. The acute physical exercise increased the muscle glycogen synthesis in all groups, with the highest values in the LF group. The other variables did not differ among the groups neither at rest nor after acute physical exercise.

## Discussion

This study evaluated the muscle glucose and protein metabolism in an experimental model using young rats fed a fructose-rich diet after fetal protein malnutrition, both at rest and after an acute bout of swimming performed at the maximal lactate steady-state intensity.

The efficiency of the low-protein diet during the fetal period in inducing the malnutrition was confirmed by the reduced values in body weight, serum glucose, total protein and albumin, corroborating previous studies using the same diet [5, 8, 9, 23]. Likewise, the diets used for nutritional recovery reestablished the circulating total protein and albumin, corroborating studies where a balanced diet induced the same effects [5, 23]. To analyze the efficiency of nutritional recovery on muscle protein metabolism after intra-uterine protein malnutrition, we performed in vitro assays of protein synthesis and degradation at the *soleus* muscle. As we expected, the rates of synthesis and protein degradation, were restored, in present study, independent of diet adopted after birth.

In short-term, fructose-rich fed groups regardless of the diet ingested during pregnancy, had a smaller body weight. In long term the fetal malnutrition seems to have a protective effect against the impairment in weight gain. This reduced body weight may be caused by fructose intolerance developed by the excessive intake of this nutrient, administered early. In a recent study, it was presented that in the suckling periods, intestinal mRNA levels of GLUT5 (the fructose transporter) and fructose transports, under normal conditions, are reduced in the rats [24]. The occurrence of symptoms originated from fructose intolerance is associated with the amount of fructose consumed [25]. In human beings, there are reports that fructose intolerance causes growth retardation [26]. Therefore, the elimination of fructose from the diet is indispensable to mitigate the harmful effects [27].

On a previous study, with an analogue experimental model [6], except for a longer period of exposure to the fructose diet (until adulthood), it was also verified that the body weight of rats fed with a fructose-rich diet was compromised. Complementary studies on this field are remarkable, since there is a lack of information in literature about the harm caused to the animal’s growth by the fructose early consumption. Previous studies that use fructose-rich diets started to administrate it during weaning [28, 29] and adulthood [30], not since the birth.

Fetal protein malnutrition followed by an elevated ingestion of fructose did not cause glucose intolerance, as there was no change in the response to glucose after its overload. These data corroborates Cambri et al. [6], using the same experimental model until adulthood. On the other hand, contradict Botezelli et al. [31] and Moura et al. [32], who detected glucose intolerance after the fructose-rich diet in older rats. These differences may have occurred as a result of age, and time of administration of the diet, and the interaction of these factors.

Studies have shown higher concentrations of liver and muscle glycogen after three weeks on a fructose-rich diet [33]. The authors justified correlates this increasing to the down regulation of the glycogen phosphorylase by the fructose-1-phosphatase. This was confirmed by Youn et al. [34] who studied the glycogen metabolism and suggested that the accumulation of liver glycogen is slightly increased by the presence of fructose, mainly by the inhibition of glycogen degradation instead of the increased synthesis, due the glycogen phosphorylase regulation by the fructose-1-phospatase. Still, in the present study, muscle glycogen was increased only in the group that ingested high amounts of fructose after a fetal protein malnutrition (LF). But this hypothesis can only be considered for the situation at rest, since, after acute exercise the glycogen was reduced in all groups. Additionally, according to the performed analyses, it is not possible to clarify the reasons why this found was not observed in the BF group.

Corroborating the present study, after an exercise bout, the muscle glycogen concentrations decreased [35–37], both in animals and humans. The observed hyperglycemic response is probably due to an increase in liver the glycogenolysis modulated by an increased sympathetic activity and/or the increased gluconeogenesis by an increasing in the secretion of glucocorticoid hormones, such as corticosterone in rats [36, 38].

The catabolic hormones lead to a breakdown of glycogen via glycogenolysis resulting in reduced muscle glycogen [39]. The intensity of the physical exercise also interfere in the glycogenolytic process, as studies indicate increased depletion of muscle glycogen at 76-80% compared at 40-48% VO_2max_ [37, 39]. In the present study, data obtained suggest increased use of muscle glycogen and lower lipids utilization, sustaining the hypothesis that a higher intensity during physical exercise is associated to an increased rate of muscle glycogen depletion, since acute physical exercise was performed at intensity equivalent to the maximal lactate steady-state. Another factor is the time of effort, since in humans, the carbohydrate metabolism predominates at 30 minutes of exercise at a 55% VO_2max_ [40]. Therefore, the exercising time used (20 minutes) in the present study favors an increased use of carbohydrates.

Galdino et al. [9] observed that malnutrition leads to alterations in the glucose metabolism, characterized by lower rates of glycogen synthesis in the *soleus* muscle in malnourished rats compared to the controls. In the present study, there were no differences, neither between the groups nor in the effect of acute physical exercise for the glucose uptake and oxidation variables by the isolated *soleus* muscle. Similar data for these parameters were observed in rats recovered from post weaning malnutrition and control groups [41], showing that fetal malnutrition did not interfere in these parameters of the muscle glucose metabolism after nutritional recovery.

Due to the depletion of muscle glycogen by acute physical exercise, we speculate that there is an increased stimulation of the synthesis of glycogen in the post exercise period, verified in the present study *in vitro*. The increased synthesis of glycogen due to the fructose-rich diet in the LF group, occurred both at rest and after a section of acute physical exercise.

Studies have shown an inverse correlation between the muscle glycogen after an acute bout exercise and the glycogen synthase enzyme [42]. Bruce et al. [35] verified an increasing in the total glycogen synthase activity immediately after an acute bout of swimming exercise in rats. Similarly, Kraniou et al. [37] observed an increase in the expression of the GLUT4 and total GLUT4 mRNA, which might contribute to the increased post exercise synthesis of glycogen. These responses were of found in efforts corresponding to 40 and 80% of VO_2max_, regardless of the differences in intensity and duration of the physical exercise [37].

Due changes in the cellular energy status, physical exercise can trigger the glucose uptake trough the 5′-AMP-activated protein kinase (AMPK) cascade leading to a GLUT-4 translocation [43–45]. Physical exercise induces a decline in skeletal muscle malonyl-CoA, which is accompanied by inactivation of acetyl-CoA carboxylase (ACC) and increased the AMPK phosphorylation (activation) [46]. So, glycogen storage has an inverse correlation with the increased pAMPK [46]. Acute exercise increases pAMPK α2 activity in red quadriceps muscle in rats and this correlated with an inactivation of ACC. And the endurance training affects the magnitude of AMPK activation by acute exercise. So, this response was greatly reduced after training, probably because of decreased metabolic stress [47]. On the other hand, contradict these founds, after acute exhaustive exercise (ultra-endurance exercise), the AMPK protein expression was increased in the trained rats and not in sedentary rats [48]. So, activity of AMPK and ACC are affected by fiber type, glycogen content, exercise intensity and exercise training [45, 47]. In the present study, these parameters about the mechanisms involved in this process were not analyzed. Future research involving these analyses can to collaborate to explain the mechanisms involved in this process. So, these are some limitations of this study.

Additionally, the amount of glycogen available is associated to the support time in a load of physical exercise. Taking this, we infer that the LF animals could sustain a longer exercise than the other animals during a prolonged exercise in maximal lactate steady-state or above this intensity. Because in these intensities occurs increased in the muscle glycogen utilization.

In summary our results showed no differences in the glucose tolerance among the groups. In addition, there were no differences in the protein synthesis and degradation. Beside that, the acute physical exercise reduced the muscle glycogen concentrations in all groups, while the LF group showed higher concentrations at rest. There was no effect of acute physical exercise upon glucose uptake and oxidation by the isolated *soleus* muscle. The glycogen synthesis was higher in the LF group at rest, and the acute physical exercise increased glycogen synthesis in all groups, reaching the highest values in the LF group. Therefore, it has observed that a fructose-rich diet administration to young rats after fetal protein malnutrition alters glycogen concentration and synthesis in the rest, but only glycogen synthesis remains altered after acute exercise. From this, it is extreme importance analyze the muscle glycogen metabolism after acute physical exercise in other intensities and times of exercise, and mainly after chronic physical exercise in this experimental model.
